# Roots compact the surrounding soil depending on the structures they encounter

**DOI:** 10.1038/s41598-019-52665-w

**Published:** 2019-11-07

**Authors:** Maik Lucas, Steffen Schlüter, Hans-Jörg Vogel, Doris Vetterlein

**Affiliations:** 1 Department of Soil Physics, Helmholtz Centre for Environmental Research –UFZ, Halle (Saale), Germany; 2 Soil Science, Martin-Luther-University Halle-Wittenberg, Halle (Saale), Germany

**Keywords:** Environmental sciences, Imaging techniques, Image processing, Patterning, Tropism

## Abstract

Contradictory evidence exists regarding whether and to which extend roots change soil structure in their vicinity. Here we attempt to reconcile disparate views allowing for the two-way interaction between soil structure and root traits, i.e. changes in soil structure due to plants and changes in root growth due to soil structure. Porosity gradients extending from the root/biopore surface into the bulk soil were investigated with X-ray µCT for undisturbed soil samples from a field chronosequence as well as for a laboratory experiment with *Zea mays* growing into three different bulk densities. An image analysis protocol was developed, which enabled a fast analysis of the large sample pool (n > 300) at a resolution of 19 µm. Lab experiment showed that growing roots only compact the surrounding soil if macroporosity is low and dominated by isolated pores. When roots can grow into a highly connected macropore system showing high connectivity the rhizosphere is more porous compared to the bulk soil. A compaction around roots/biopores in the field chronosequence was only observed in combination with high root/biopore length densities. We conclude that roots compact the rhizosphere only if the initial soil structure does not offer a sufficient volume of well-connected macropores.

## Introduction

Growing roots have to overcome soil mechanical resistance imposed by soil structure^1^,so that compacted soils may limit root growth and plant yield^2–4^. The availability of macropores and their connectivity are therefore often stated to be a limiting factor in plant growth, since roots preferentially grow into existing macropores and by this can bypass zones of high mechanical resistance^3,5^. This influence of soil structure on root growth is often in the focus of research.

However, the interactions of plant roots and soil structure are two-way, i.e. there is also an effect of plant roots on soil structure. During soil exploration, roots push through the soil and alter physical, chemical and biological properties in their vicinity, the rhizosphere^6^. These alterations may persist after roots are degraded, leaving behind a dense system of connected biopores^7^ These biopores, now an integral part of soil structure, in turn feedback on root growth providing pathways of low mechanical resistivity with wall properties reflecting former root activity and in part activity of soil fauna.

At the system scale root derived biopores enhance soil structural properties like air permeability, rootability and saturated water flow^8^. At the local scale of the rhizosphere, root induced structural changes are not only relevant for microbial habitat quality, but in particular for transport of water and nutrients. The rhizosphere is the bottleneck water and nutrients have to pass before entering the root. Likewise, substances released by the plant will diffuse through this zone. Aravena *et al*.^9,10^ have illustrated that it is crucial to define and describe changes in rhizosphere porosity in order to model rhizosphere processes, as an enhanced rhizosphere compaction (decreased porosity) had a positive effect on root water uptake.

Since root diameters are often larger than existing pores, growing roots are believed to compact the rhizosphere^9,11,12^. Dexter first modeled this compaction. His model describes the exponential decrease of porosity toward the roots, defined by a constant multiple of the root diameter.

More recently, different imaging techniques enabled direct *in situ* visualization of the root-soil interface and the soil structure gradient extending into the bulk soil^13–16^. Vollness *et al*.^17^ confirmed for very young *Zea mays* that the compaction around primary roots in sand is exponential and depends on the root radius. However, some of these studies revealed contradictory results, i.e. increase of porosity in the rhizosphere^13,15,16,18^. Feeney *et al*.^16^, analyzed not a gradient but aggregates from the rhizosphere in comparison to aggregates from an area accessible only by hyphae and a control without plants using X-ray CT with a resolution of 4.4 µm. Their results showed a significant increase in the porosity of the rhizosphere compared to bulk soil. Helliwell *et al*.^13^ referred the increase in porosity they observed to processes like shrinking and swelling, which are enhanced in the rhizosphere. In a subsequent work^18^ they showed that the porosity (density) gradients depend on plant species, texture and distance to the root surface. The latter was also observed by Koebernick *et al*.^19^, describing high porosity directly at the vicinity of the epidermis of roots which grew in packings of 2 mm aggregates. They assigned this to a ‘surface/wall effect’, i.e. the loose packing of particles against large surfaces described by Suzuki *et al*.^20^. Therefore, Koebernick *et al*.^19^ added this ‘surface/wall effect’, i.e. the packing geometry of spherical soil particles, to the model of Dexter^12^. In addition to the formation of new root channels, roots can also penetrate the soil by using existing pores or cracks^1^.

In this manuscript, we reconcile the disparate views on porosity changes in the rhizosphere by: (1) considering the gradient extending from the root surface into the soil with high spatial resolution, i.e. allowing for a differentiation between the zone immediately at the root-soil interface (0–100 µm) and the zone at slightly larger distance (up to 1 mm) and by (2) addressing the interplay between porosity and pore size distribution on the one hand, and root length distribution across different root diameter classes on the other hand and by (3) relating porosity changes in the rhizosphere to the antecedent soil structure prevailing before roots penetrated the soil.

We hypothesize that root induced compaction described by Dexter^12^ depends on soil pore characteristics and decreases with the amount of connected macropores, since roots which grow into existing macropores do not necessarily have to align soil particles as they move through the soil. We conducted an experiment in the climate chamber with *Zea mays* and three different bulk densities to observe the impact of macropore characteristics on root induced compaction and transferred this to existing field information on biopores in undisturbed field samples from Lucas *et al*.^7^.

Lucas *et al*.^7^ described the formation of biopores using more than 250 samples for X-ray CT from a space-for-time chronosequence, including six time points of soil development in two different depths.

## Methods

### Laboratory experiment on soil compaction

Soil for laboratory experiment on soil compaction was taken from a reclamation area at the Garzweiler open pit mine (Germany) described in Pihlap *et al*.^21^. The soil was homogenized, passed through a 2 mm sieve and air-dried (~2% gravimetric water content). A basal fertilizer application consisting of 50 mg N kg^−1^, 50 mg K kg^−1^, 40 mg P kg^−1^ and 25 mg Mg kg^−1^ was applied in the form of NH_4_NO_3_, K_2_SO_4_, MgCl_2_ × 6H_2_O and CaHPO_4_, respectively.

Five replicates were packed for three different bulk densities (1.30 g cm^−3^, 1.45 g cm^−3^, 1.60 g cm^−3^). The columns (7 cm $\varnothing$ and 25 cm height) were filled up to 23 cm and a small cylinder (2.5 cm $\varnothing$, 4 cm height) was placed in the center of the surface area. These cylinders were packed with soil of 1.3 g cm^−3^ and the maize seeds (*Zea mays var*. B73; one seed per column) were placed within this cylinder to ensure a good germination in all treatments and to force the seminal roots to grow at a steeper angle; i.e. avoiding growth against the cylinder wall early on. The seeds were previously surface sterilized in 10% H_2_O_2_ for 10 minutes, washed with deionized water and then placed 2 hours in a solution of 2 mM CaSO_4_. The entire soil surface was covered with a 2 cm thick layer of quartz gravel to reduce evaporation. The columns were watered from the top and bottom to a water content of 30 Vol-% and a 30-μm nylon mesh enabled re-watering from the bottom twice a week. The plants grew for 20 days in a climate chamber under following conditions: Relative humidity of 65%, 12 h day at 22 °C with 350 μmol m^−2^ s^−1^ PAR and a night temperature of 18 °C. During harvest, shoot fresh and dry weight (24 h in an oven at 65 °C) was determined. The plant samples were ground and C, N were analysed using a coupled system of elementar analyser and quadrupole mass spectrometer (Vario EL cube, Elementar Hanau, Germany; Quadropole MS ESD 100, ICI Bremen, Germany). After acid digestion ICP-OES analyses were conducted for selected plant nutrients P, K, Ca, Mg, Mn, Fe.

Undisturbed subsamples (3 cm $\varnothing$; 3 cm height) were taken in three different depths (7–10 cm, 12–15 cm, 17–20 cm) with a subsampling device (UGT GmbH, Germany).

### Chronosequence

An existing X-ray µCT-dataset from Lucas *et al*.^7^ was used to investigate root induced compaction in the field. The chronosequence and sampling approach is described in detail in Lucas *et al*.^7^ and Pihlap *et al*.^21^. Briefly, the plots of the space-for-time chronosequence were formed by a standardized reclamation technique after lignite mining, i.e. they were all formed on the same homogenized initial substrate (unweathered loess, weak alkaline pH, high CaCO3 concentration). Samples were taken on three plots and two depths (0–20 cm and 40–60 cm) from sites in six different age groups 0, 1, 3 years (Lucerne sites, no tillage) and 6, 12 and 24 years (cereal sites, tilled) after first sowing. Three undisturbed samples (20 cm height, 10 cm $\varnothing$) were taken per plot with a tailor-made drill for undisturbed sampling of cylindrical soil cores (UGT GmbH, Germany) and later three subsamples (3 cm height, 3 cm $\varnothing$) per sample were taken. The biopores in these samples were mainly formed by roots^7^, which enables a comparison to the pot experiment.

### X-ray microtomography

Differences in root-induced compaction between the three different treatments of the pot experiment were evaluated by scanning the 45 cylindrical subsamples of 3 cm $\varnothing$ (5 replicates, 3 depths). Differences in visible porosity (limited by the resolution of 19 µm) and grey scale gradients extending from biopore walls in field samples were described based on 279 subsamples with a diameter of 3 cm $\varnothing$ (6 fields, 2 depths, 3 plots, 9 replicates). The 0-year old field at the second depth and one plot of the 3-year old field were not evaluated^7^. All samples were scanned with a X-ray microtomograph (X-TEk XCT 225, Nikon 162 Metrology) with an Elmer-Perkin 1620 detector panel (1750 × 2000 pixels) using 130 kev/150 µA and reconstructed with an spatial resolution of 19 µm as explained in more detail in Lucas *et al*.^7^.

3D non-local means filter (UnbiasedNonLocalMeans in ITK, Revision 1.21^22^) was used for noise removal. The images were previously converted to 16-bit and the value 1000 was added, to reduce the effect of the non-linear transformation of gray values by this filter in dark areas. The images of the Lab experiment were previously inverted to maintain a high contrast between roots and pores. After filtering, the images were reduced to 8-bit and an unsharp mask (radius 2 and mask weight 0.2) was applied using Fiji.

For the threshold detection of pores (including roots) 5 different segmentation methods were applied as described in Schlüter *et al*. (2014), then Hysteresis-Thresholding of the 3D segmentation plugin (version V3.83) was applied in Fiji^23^. The latter enabled us to segment all pores, regardless of whether they were filled with air, water or roots. The pore size distribution (PSD) was calculated by local thickness method in Fiji (version 1.4.6), which uses the maximum inscribed ball method. In addition, the volumetric Euler number was also calculated with the MorphoLibJ plugin in Fiji (version 1.3.3^24^) to characterize pore topology. High negative values indicate a high number of connections between pores. Incorrect classifications and noise strongly influence the Euler number^25^, therefore a pore opening was performed before the calculation and pores smaller than or equal to four pixels (76 µm) were removed.

The Rootine Protocol^26^ was used and adapted to segment all tubular biopores and roots. This protocol uses the Tubeness plugin on Fiji to separate tubular objects from the remaining irregularly shaped pore network. It is combined with a scale-space approach, i.e. convolution with a Gaussian blur of different standard deviations σ are used to detect tubes of different diameters. The number of different σ’s in the scale space was different between pots (four) and field soil (eight) with a wider range of biopore diameters. A combination of Connected Components Labeling and Label Size Opening implemented in the MorphoLibJ plugin in Fiji (Version 1.3.3, Legland *et al*., 2016) filtered smaller misidentified pores out. Due to the tubular shape of the segmented roots and biopores, the local thickness method in Fiji could be used to obtain information about the biopore diameter classes and corresponding length. More details on images processing and analysis of 3 cm $\varnothing$ cylinders can be found in Lucas *et al*.^7^.

### Evaluation of gradients around biopores and roots

**Figure 1:**
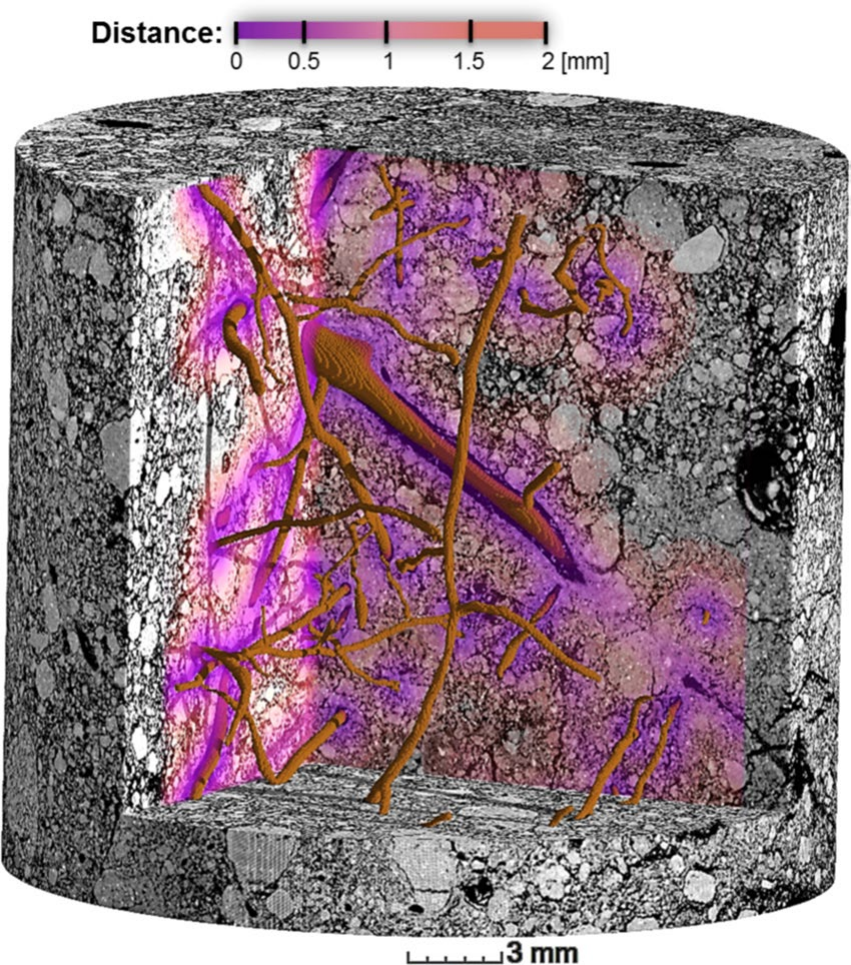
3D visualization of a RGB image created to analyze density gradients with distance to the biopore. The three channels of the RGB image contain 8-bit information on gray values, pores and the distance map (gradient color), which shows the distance from every pixel to the next biopore (brown).

A new protocol was developed to analyse root induced compaction by combining the images of filtered gray values, visible porosity, and euclidean distances into the different channels of a RGB-image (Fig. 1). The distances were was calculated using euclidean distance transform (EDT) in Fiji, which describes the Euclidean distances from all soil voxels to the nearest biopore/root. The resulting RGB image was analyzed by calculating the average porosity and average gray value as a function of distance. Every fifth voxel within a loop through all slices was read out to reduce computational time.

**Figure 2:**
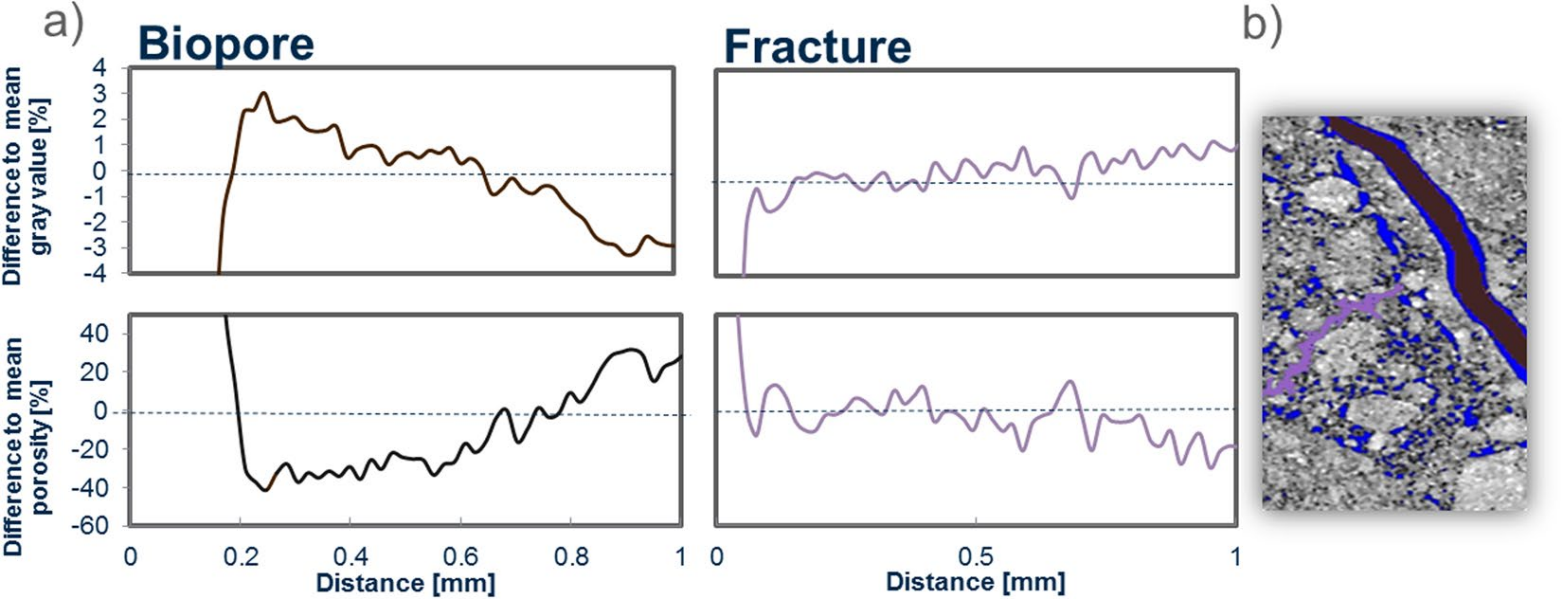
(**a**) average of gray values and porosity (blue) as a function of distance to the biopore (brown) and the fracture (purple) of the image left in the 2D section (**b**).

The changes in visible porosity of one biopore show a clear compaction extending up to 0.6 mm into the rhizosphere, whereas this compaction is absent around a fracture (Fig. 2a). In contrast, the direct vicinity of this segmented biopore shows low porosity. The main reason for this is the roughness of the pore wall since the Tubeness filter describes the biggest tube “fitting” into the pore, which excludes the porosity along the rough pore walls (Fig. 2b)^7^. Therefore, high roughness of the pore wall increases the distance at which a possible compaction could be detected^19^. Changes in gray values are inverse to porosity and show almost the same trend. This agreement between gray values and visible porosity indicates that image processing did not affect the changes of gray values with distance. These changes are closely related to changes in bulk density. In contrast, visible porosity is limited by image resolution and shows therefore higher local variability. Therefore, only the gray value results will be shown in the remainder of this manuscript and corresponding changes in visible porosity can be found in the supplement.

In order to describe changes around roots of different size classes a Local thickness analysis was conducted on the segmented root/biopore images and the outcome was thresholded at a diameter value of 13 pixels. By this, Euclidean distances could computed separately for roots/biopores >250 µm and <250 µm. To reduce the effect of the other root class, corresponding root images were subtracted from the Euclidean distance images, e.g. the porosity and gray value of the roots >250 µm were not taken into account for the analyses of the compaction around roots <250 µm. The roots >250 µm were extended with five 3D dilatation steps before they were subtracted from the EDT map of the roots <250 µm and vice versa to exclude the potential gradients associated with the other root diameter class.

### Statistical analysis

For the pot experiment conducted in the climate chamber, standard errors and mean values of five replicates are provided. The chronosequence is described by the standard deviation and mean values of 3 plots for each year and depth in which each plot is represented by an average of the 9 technical replicates (i.e. X-ray CT scans). A one-factorial ANOVA in conjunction with Tukey’s HSD test revealed significant differences in the data. A log-transformation was used for porosity data prior to statistical analyses. A Kruskal-Wallis test followed by Dunn’s multiple comparisons test was used to reveal differences within the highly non-linear Euler number. For all statistical analysis the software R 3.53 and the package agricolae^27^ was used.

## Results

### Pot experiment

**Figure 3:**
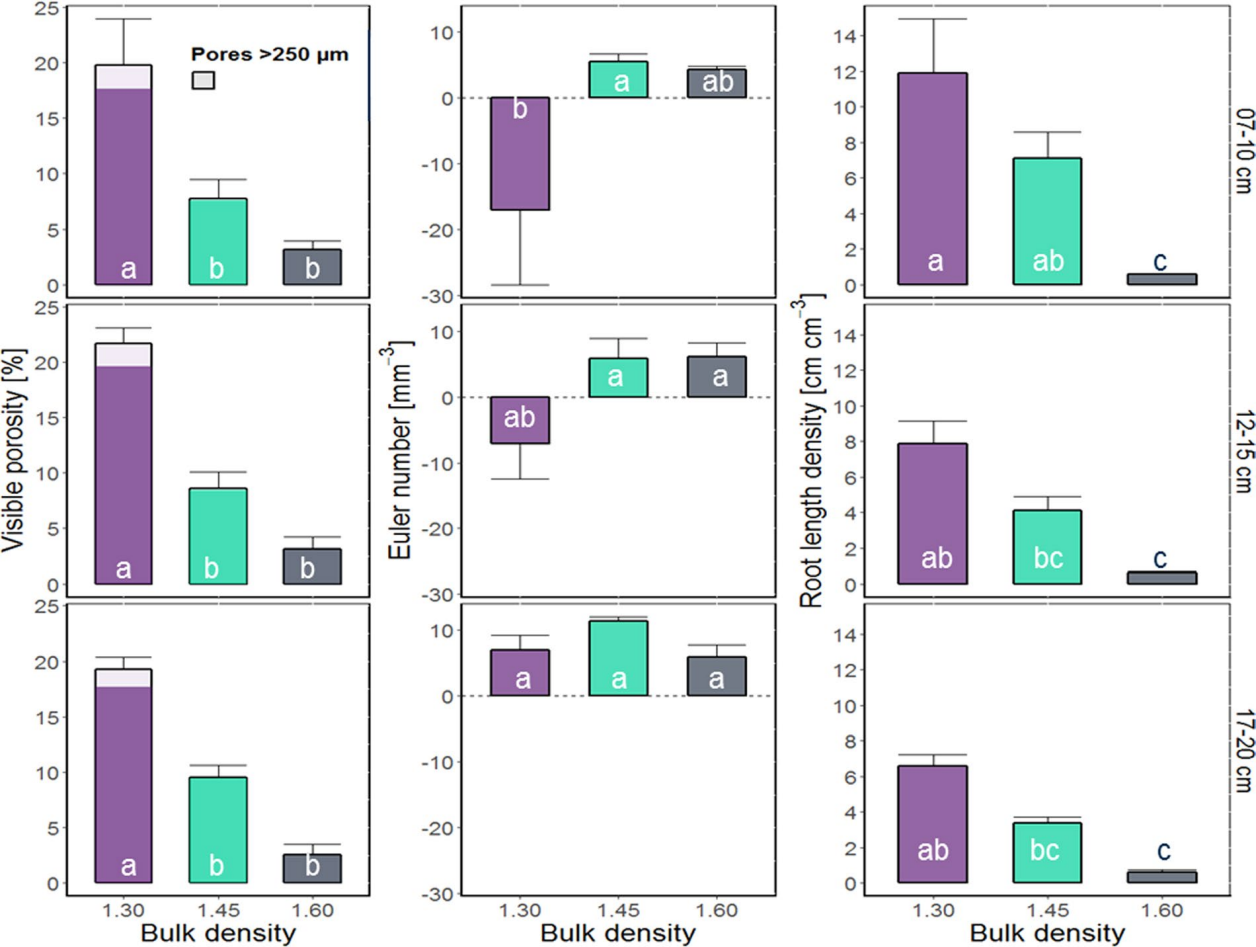
Mean values and standard errors (whiskers) of visible porosity, Euler number and root length density for each of the three different treatments (bulk density of 1.30, 1.45, and 1.60 g cm^−3^) and depth within column (7–10 cm, 12–15 cm, 17–20 cm) derived from µCT-scans of 3 cm $\varnothing$ samples. Visible porosity shows pores >38 µm (two voxels), the Euler number shows the connectivity of pores >76 µm. Different letters indicate significant differences (n = 5). Inset shows the subsampling scheme.

**Figure 4:**
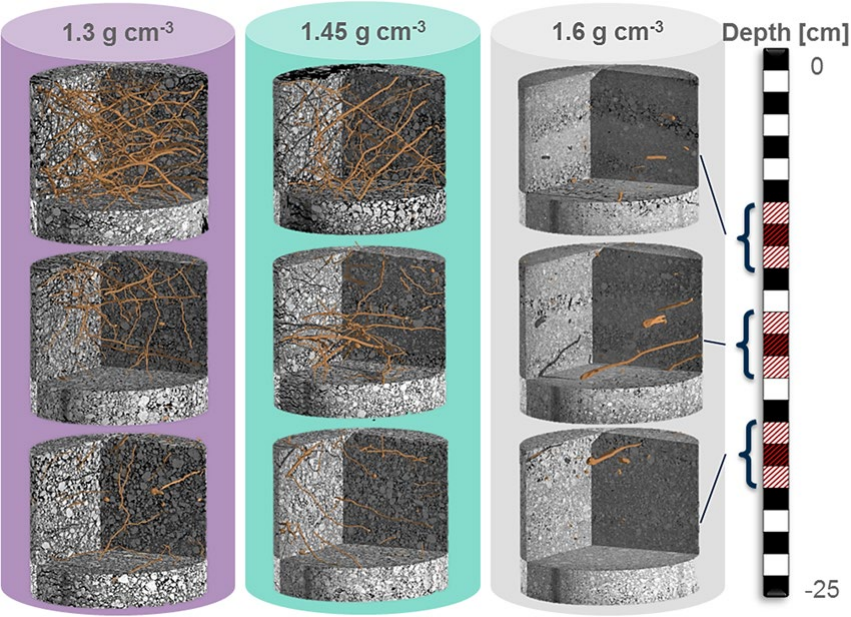
3D visualization of roots in representative 3 cm $\varnothing$ subsamples for each of the three different treatments (bulk density of 1.30, 1.45, and 1.60 g cm^−3^) and depth within column (7–10 cm, 12–15 cm, 17–20 cm).

Visible porosity decreased significantly with increasing bulk density (Fig. 3). The effect of compaction was especially high for macropores, i.e. pores >250 µm were almost completely absent in samples with bulk densities of 1.45 and 1.60 g cm^−3^. Shoot growth decreased significantly with increasing bulk density (Table S1). Likewise root growth (Figs 3 and 4), decreased significantly with increasing bulk density, on top of an expected decrease with depth. The Euler number, a measure for the connectivity of the pore system, revealed low connectivity (positive values) in relation to isolated macropores for almost all treatments and depth (Fig. 3). With increasing root length density the Euler number decreased (negative values in the first two depths within the treatment of 1.30 g cm^−3^), i.e. numbers of pore connections are higher than the number of non-connected pores.

The frequency distribution of the Euclidean distances from soil voxels to the next root (Fig. S1) showed a clear shift towards smaller distances with increasing porosity (decreasing bulk density) for all sampled depths of the columns.

**Figure 5:**
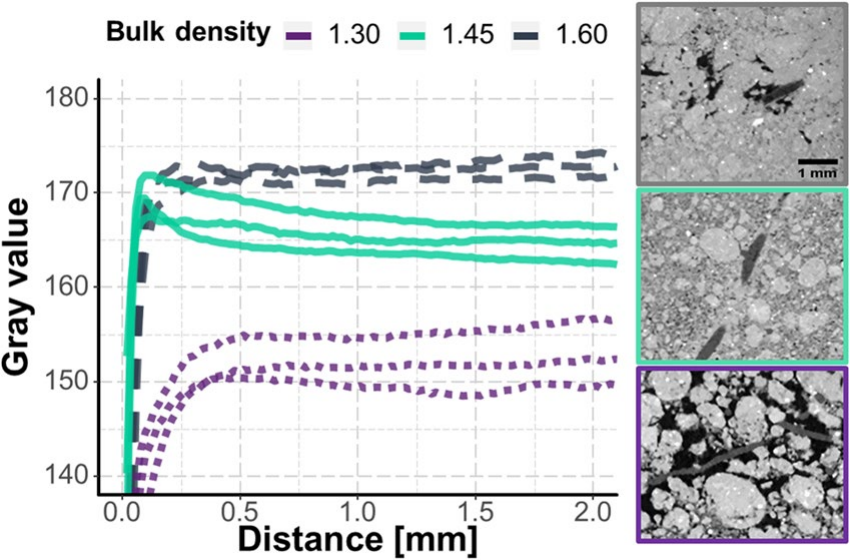
Change of gray values with distance to the roots for three random subsamples for each of the three treatments (bulk density of 1.30, 1.45, and 1.60 g cm^−3^) and corresponding µCT slices showing roots within the different compacted soils.

**Figure 6:**
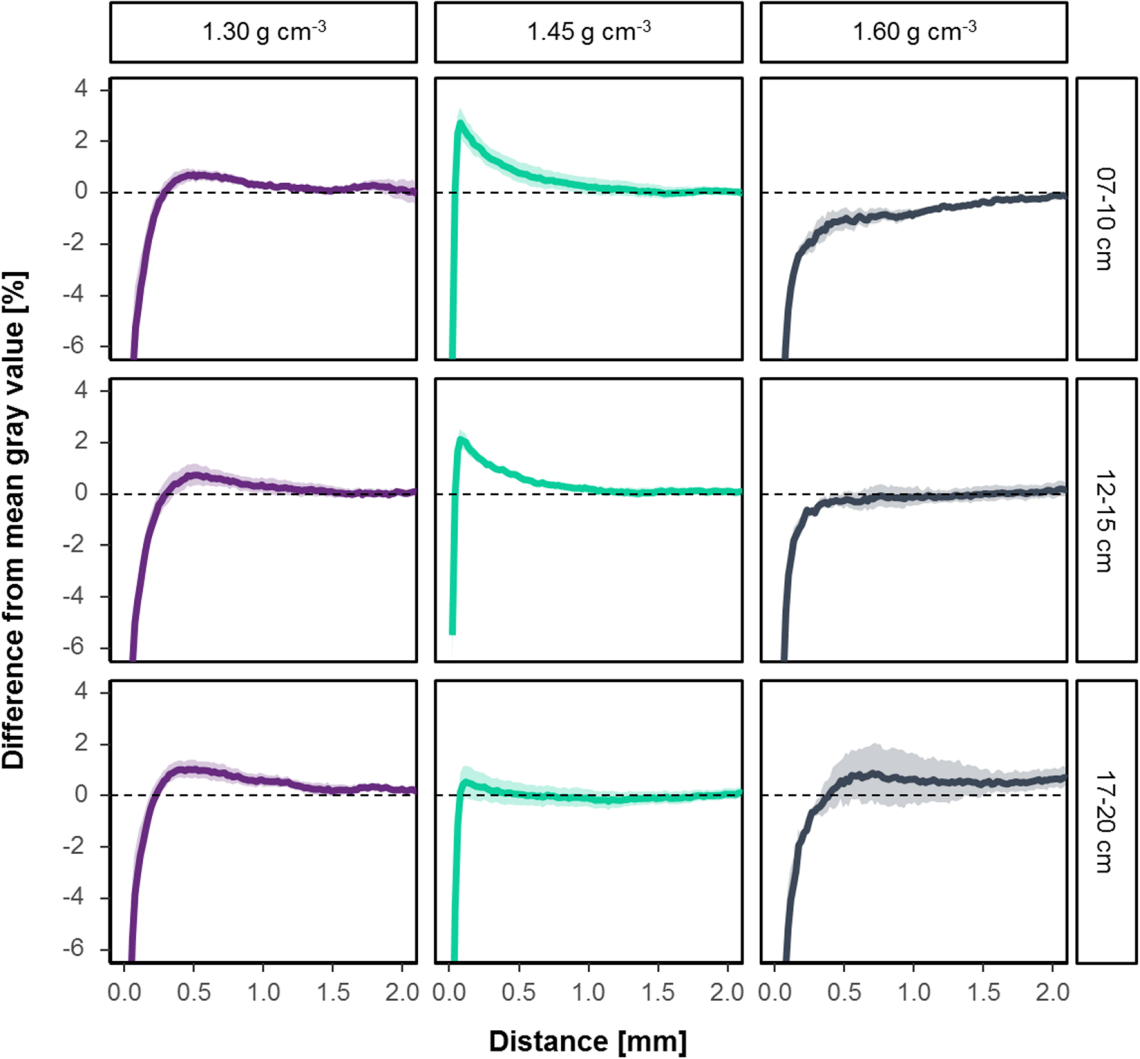
Mean change in gray value with the distance to the root surface relative to the mean gray value of a sample. Shadows indicate the standard errors (n = 5).

As expected, the gray values in the bulk soil, i.e. the distance from the root >1.5 mm, reflected the bulk densities of randomly chosen samples of the three treatments (Fig. 5). The mean transects of gray values with increasing distance from the root surface differ between treatments. By normalising the data, these differences can be seen more clearly. This was achieved by dividing the data by the mean gray value of the respective sample (Fig. 6). Up to a distance to the roots of about 0.1 mm (5 pixels), the gray values are strongly reduced in all samples. Beyond this distance, gray values for the treatment with the intermediate bulk density (1.45 g cm^−3^) increase above the reference value for the bulk soil and flatten out as distance to the root surface increases further. For the treatment with bulk density of 1.30 g cm^−3^, the overshoot in normalized gray value, i.e. positive difference indicating compaction, is not as pronounced but still present. For the bulk density of 1.60 g cm^−3^ no such peak is visible.

In summary, the highest root induced compaction was detected in the samples of intermediate bulk density (Fig. 6). This effect reached up to 1.5 mm into soil, but there was a decreasing trend with increased depth. For the treatment with lowest bulk density, a zone with even lower bulk density in the direct vicinity to the root surface is followed by a slightly compacted zone at distances larger than about 250 µm. It should be noted that this reflects the mean transect within a sample calculated for a number of roots and that individual transects can have very different characteristics.

Figure S2 reveals that the mean change in visible porosity with the distance to the root surface has the same trend as the changes of gray values but standard error is generally larger. The visible porosity of the highly compacted rhizospheres in the intermediate bulk densities decreased by up to 30%. For the first depth of the treatment with a bulk density of 1.45 g cm^−3^ the model of Dexter and the extension of Koebernick *et al*.^19^ are in line with the data. The fitted model of Koebernick *et al*.^19^ resulted in a particle diameter of 1.974 mm, which corresponds to the 2 mm sieving. Therefore this curve can be seen as what one would have expected. However, all other curves show trends like those of the gray values.

**Figure 7:**
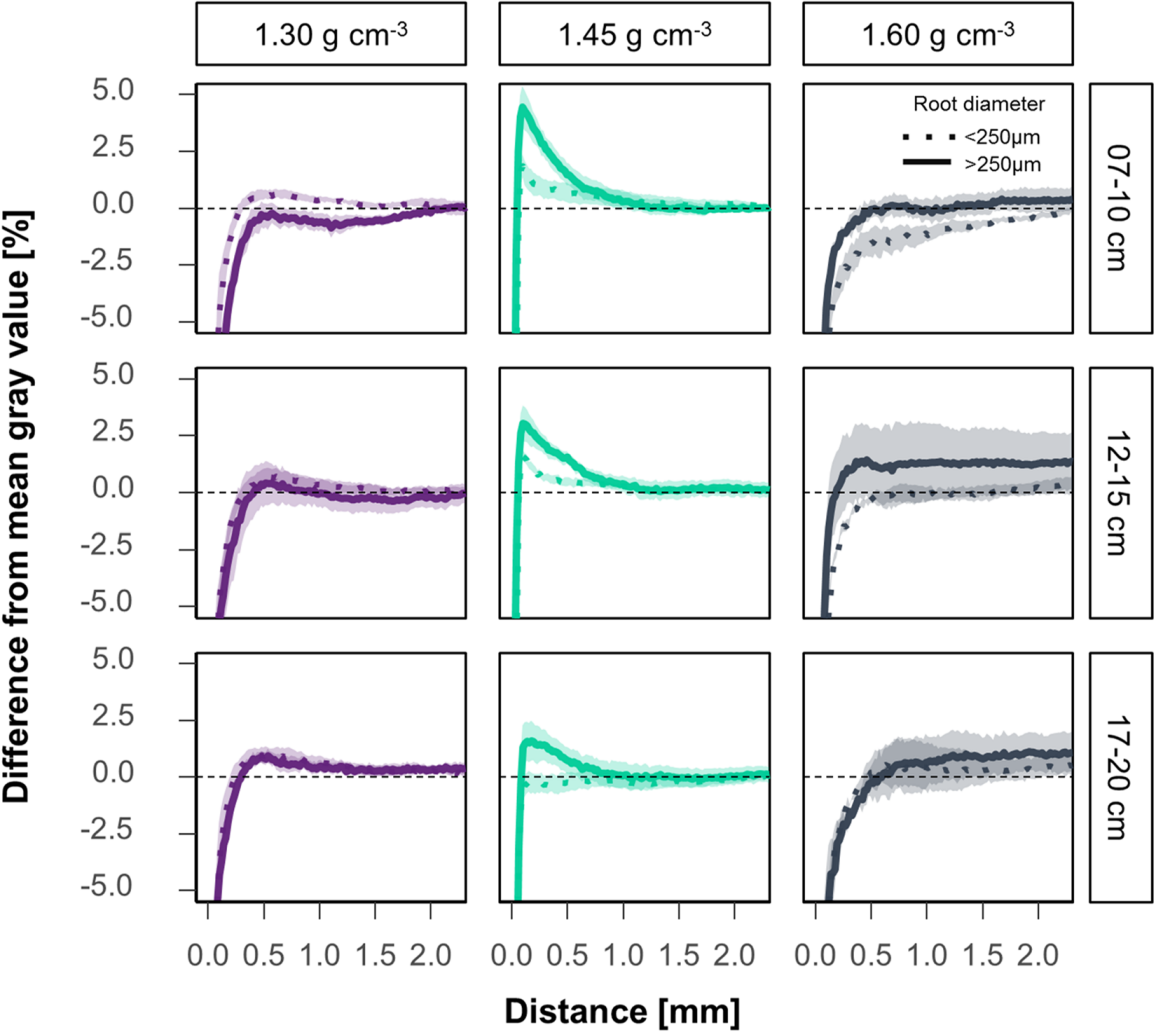
Mean change in gray value with the distance to fine and coarse roots relative to the mean gray value of a sample. Dotted lines represent the mean changes around roots smaller 250 µm and solid lines these for roots with diameters greater than 250 µm. Shadows indicate the standard errors (n = 5).

Analyzing the results separately for roots >250 µm and those <250 µm clearly showed that compaction at a distance >100 µm is more likely to occur for larger root diameters at an intermediate bulk density (Fig. 7). The compaction in a distance >250 µm of the samples in the first depth with a bulk density of 1.30 g cm^−3^ is here the exception to the rule. Again, results expressed for visible porosity instead of gray values followed the same trend (Fig. S3).

**Figure 8:**
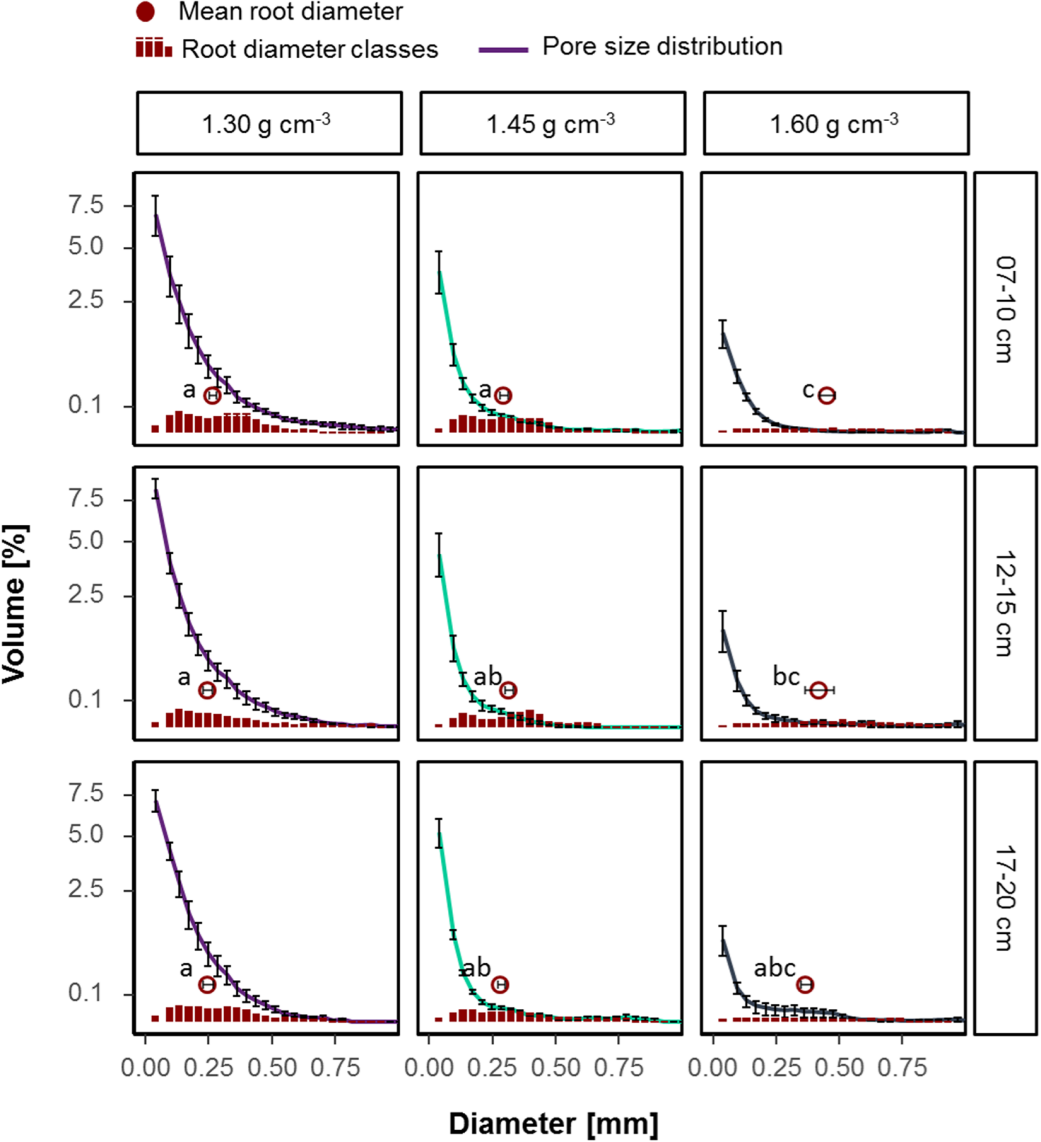
Mean volume of root and pore diameter classes for each of the three different treatments (bulk density of 1.30, 1.45 and 1.60 g cm^−3^) and depth within column (7–10 cm, 12–15 cm, 17–20 cm). Different letters indicate significant differences between mean root diameters (red circles). Whiskers show the standard error (n = 5).

The comparison of pore size distribution and root diameter classes based on volume (Fig. 8) illustrates the fraction of pores occupied by roots. Since roots are treated as pores during segmentation, the root classes must be a subset of the pore classes. The difference between the diameter classes can thus be regarded as empty macropore space, i.e. pores not filled with roots. For the lowest bulk density (1.30 g cm^−3^) there is still a large share of empty macropores, while in the other treatments only pores smaller than 250 µm were empty. Increasing bulk density led to significant increase in mean root diameters.

### Chronosequence

The bulk densities in the field (same substrate as pot experiment) were in the range of 1.40–1.61 g cm^−3^ (Table 1)^21^. Visible porosity was at no time less than 5%, in contrast to samples repacked to 1.60 g cm^−3^ in the lab experiment. The Euler number was negative on all fields and especially low in the depth of 40–60 cm^7^.

**Table 1:** Mean values and standard error of bulk density, Euler number (connectivity) and visible porosity of the chronosequence. n = 3 plots. Bulk density data from Pihlap *et al*. 2019 (mean values per plot for 1–5 and 15–20 cm depth and 41–45 cm), Euler number from Lucas *et al*.^7^.

Depth	Field	Bulk density [g cm^−3^]		Euler number [mm^−3^]		Visible porosity [Vol-%]	
0–20 cm	L0	1.54	±0.00	−8.0	±1.9	6.81	±1.46
	L1	1.61	±0.01	−3.3	±0.4	5.39	±0.55
	L3	1.61	±0.00	−7.8	±2.5	7.59	±0.67
	B6	1.46	±0.02	−5.1	±1.3	11.83	±1.53
	W12	1.50	±0.01	−5.9	±3.2	11.55	±3.73
	W24	1.55	±0.02	−6.8	±1.2	12.17	±3.45
40–60 cm	L1	1.66	±0.02	−18.9	±10.8	13.26	±3.04
	L3	1.40	±0.02	−14.4	±7.1	9.61	±2.18
	B6	1.63	±0.03	−13.3	±0.6	10.43	±1.85
	W12	1.50	±0.04	−12.4	±3.0	10.44	±2.17
	W24	1.52	±0.02	−12.5	±7.1	10.48	±3.37

While the biopore densities on the chronosequence increased over time, there were only minor changes in the pore size distribution^7^. This led to a decrease in the fraction of macropores, which are not biopores. Thus, macropores are transformed to biopores with time (Fig. S4). There was a trend towards induced compaction of the rhizosphere/biopore wall over time too, especially in the second depth (40–60 cm), which was not affected by soil tillage (Fig. S5). However, the vicinity of the biopores on the 24-year-old field in 40–60 cm depth was less compacted compared to samples from the 12-year-old field. Samples of tilled layers (6, 12, and 24 years after reclamation) were characterized by high standard deviations, i.e. some biopores of the samples show a high rate of compaction, while the vicinity of others is highly porous (Fig. S5).

**Figure 9:**
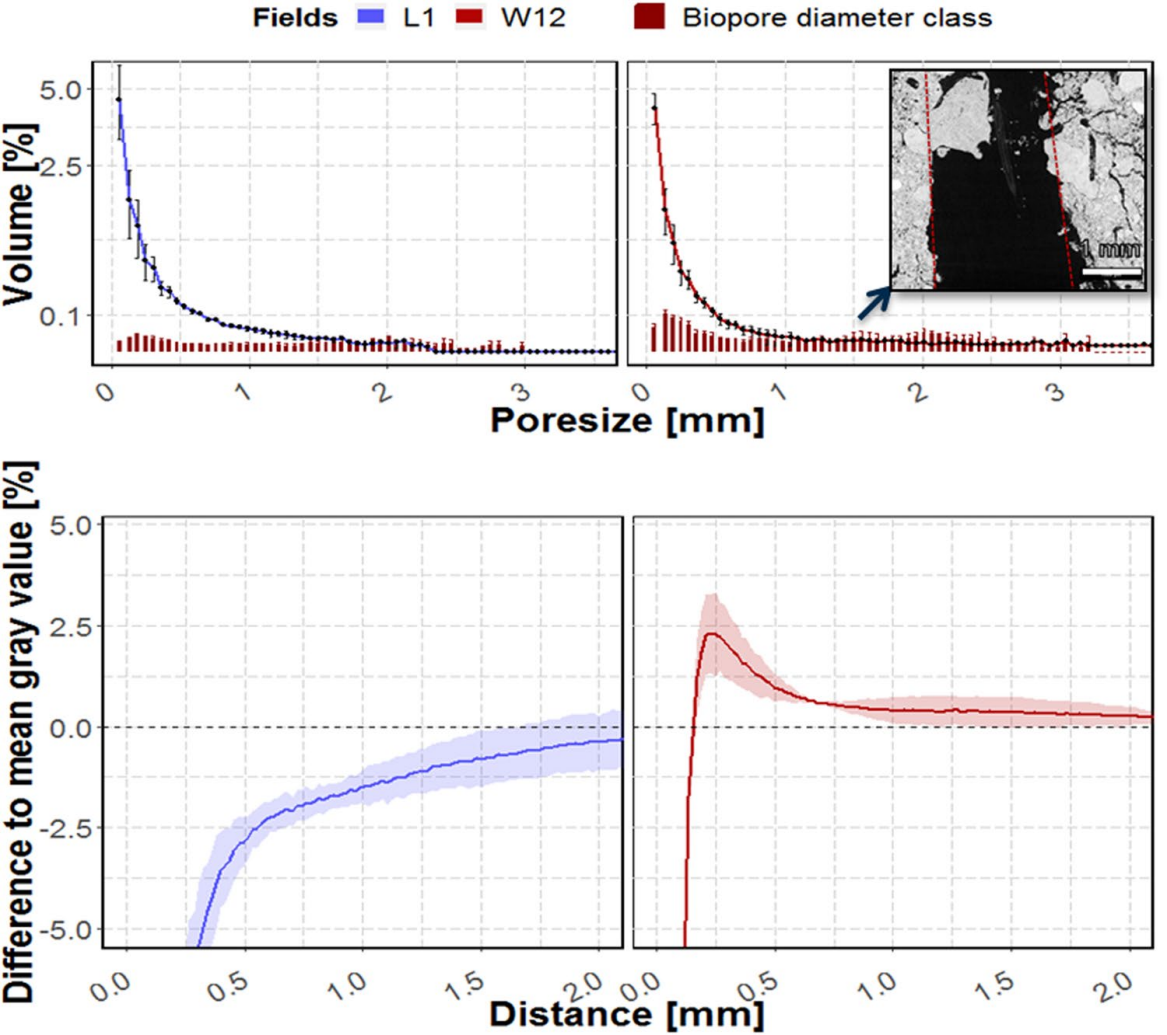
Mean volume of biopore and pore diameter classes (upper graphs) and change in gray value with the distance to the biopore surface relative to the mean gray value of a sample (lower part). Samples are taken on a 1 year old field (blue) and a 12 year old field in 40–60 cm depth on the chronosequence. Inset shows a filled biopore and how the tubeness filter would assign the borders of this biopore (red line). Shadows and whiskers indicate the standard deviation (n = 3 plots, which in turn each are determined by the result of 9 µCT scans).

Thus, samples from 40–60 cm depth of the 1-year and 12-year old field (Fig. 9) can be regarded as “endmembers”. The 1-year old field, characterized by a high amount of macropores, in relation to biopores, and highly porous biopore walls in contrast to the 12-year old field, containing macropores which are mostly biopores and which show a high compaction rate in their vicinity. For diameter classes >1 mm the biopore volume occasionally exceeded the volume of pores, although the biopores should be a subset of all visible pores. That is because the Tubeness filter also detects biopores filled by smaller particles, while the pore size distribution does not (Fig. 9, inset).

## Discussion

### The effect of soil compaction on plant roots

In line with previous work^28^ increasing bulk density decreased root length densities and plant growth above ground and led to an increase in mean root diameter (Fig. 8). The increase in bulk density to 1.60 g cm^−3^ at the given water content lead to mechanical impedance that was too high for the maize roots to elongate. The elongation of individual roots is mostly limited by the mechanical impedance of the soil^1,29–31^. The decrease in root length density increased the Euclidean distances between roots (Fig. S1). Therefore, plants of this treatment were not able to cover their nutrient demand, illustrated for phosphorus in the supplement (Table S1).

**Figure 10:**
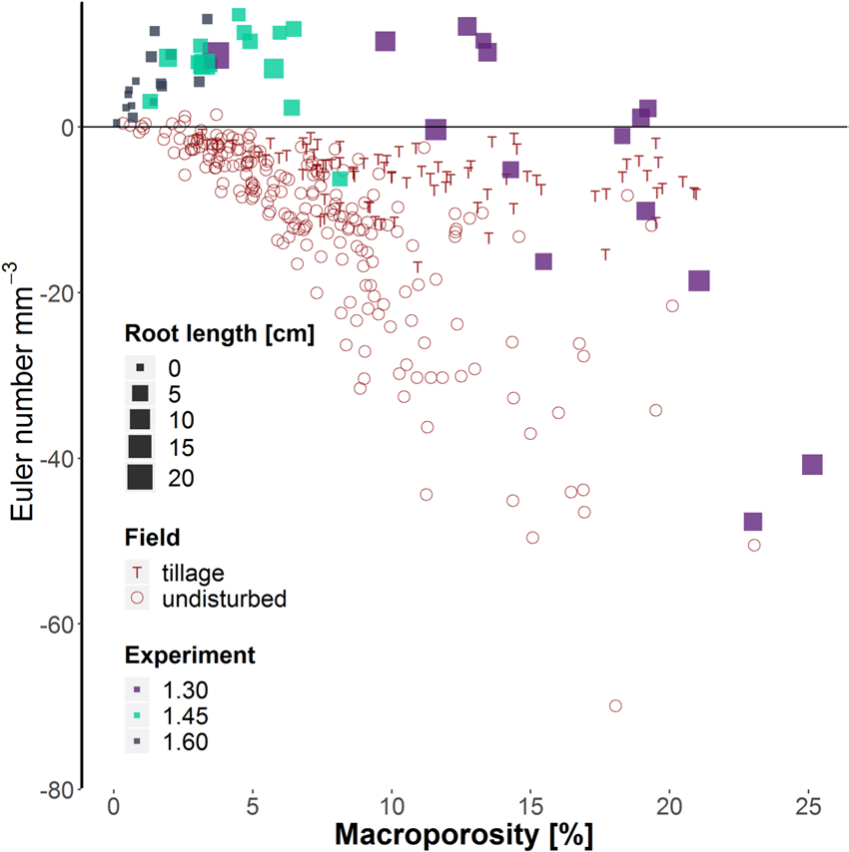
Relationship between connectivity (Euler Number) and visible porosity for 3 cm $\varnothing$ field samples (red) and the different treatments of the Lab experiment. Low values of the Euler Number reveal a high amount of connections/redundant loops in the pore system (>76 µm). The root length densities of the lab experiment are represented by the size of the squares.

However, bulk density alone is not adequate to describe changes in soil structure^21,32^. Air-filled porosity was calculated to be more than 10% for the pot experiment, which is regarded as a critical value for plant growth. However, an increase in compaction can result in much smaller equivalent pore diameters. This affects air permeability, which is correlated with pore continuity and pore sizes^4^. The fact that high bulk densities (>1.60 g cm^−3^) did not limit root growth in the field^7^, but in the repacked soil, may be explained by a completely different pore structure created during packing of the soil columns. A highly connected pore system (especially macropores >root diameter) enables roots to bypass zones of high mechanical impedance in the field^3,5,33–35^, but did not exist in the pots. The only roots that could grow in the dense samples in the pot experiment were therefore found in areas with macropores that were not compacted during packing (Fig. 4). Under wet conditions, penetration resistance of the soil decreases, but oxygen supply in these dense soil can become limiting^36^. The connectivity (Euler number) of the macropores is therefore a key parameter defining plant growth. Figure 10 reveals strong differences in the connectivity of field samples and the lab experiment. All field samples, especially those, which are not directly affected by tillage, are characterized by a high number of connected macropores (negative values). In contrast, repacked soil samples show much higher Euler numbers at the same visible porosity. Soil aeration and the probability of growing roots to find existing macropores was therefore especially low for the dense repacked soil samples in the pot experiment, characterized by a low number of unconnected macropores. Our findings highlight therefore the importance of connected macropores for plant growth by enabling easier access to deeper soil layers and thus reducing the impact of soil compaction.

### Root induced compaction

**Figure 11:**
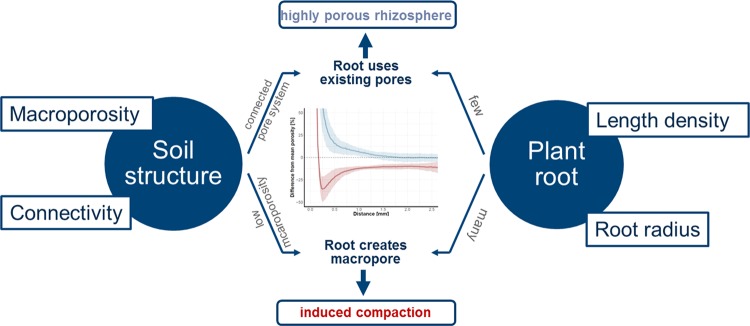
Mechanisms of root induced compaction.

Our work reconciles contradictory results from previous studies where on the one hand an increase in porosity in the rhizosphere was described^13–16^ or root induced compaction was measured^9,11,19^. These contrasting results can be integrated by merging (a) the mechanistic physical effect of root growing into the soil pushing particles to the side and inducing compaction by creation of new pores, and (b) plant interaction with existing soil pore structure, i.e. root growing in existing macropores with larger diameters than the root itself (Fig. 11). This general interaction between growing roots and soil structure should not be seen as the only process that influences root-induced compaction. However, our results showed that the initial soil structure is an important factor in predicting root-induced compaction alongside others as soil texture, drying cycles and plant species^18^.

Although we partly include sub-resolution pores through the analysis of gray values, it can be assumed that not all changes, e.g. induced by root hairs^14^, are covered by our analyses. In all samples we found an increased porosity directly next to the exodermis (distance <100 µm). These findings are in line with various studies with high resolution^13,14,18,19^. Helliwell *et al*.^18^ observed changes in porosity with distance to the root of three different plants in a loamy sand and a clay loam with bulk densities of 1.20 and 1.50. They found a significant interaction of changes in porosity with bulk density, plant and texture. They mainly accounted cracks formed by shrinking and swelling processes and gap formation for the increase of porosity in the direct vicinity of the root exodermis. A visual assessment of our images revealed only a low number of this cracks typically formed radially around roots. This processes therefore only played a minor role in the increased porosity around roots. In addition, Koebernick *et al*.^19^ found no significant influence of moisture conditions (wet vs wet and dry) on pore structure around roots. The fitted model of Koebernick *et al*.^19^ resulted in a particle size of around 2 mm which is in line with our sieving procedure. Therefore we can assign the increased porosity directly next to the exodermis (distance <100 µm) to the surface/wall effect described in Koebernick *et al*.^19^.

However, samples of 1.30 g cm^−3^ showed a decreased porosity up to a distance of around 250 µm and the rate of compaction decreased with depth in samples from the intermediate bulk density (Fig. 6). There was also a tendency of less compacted rhizospheres in the high-density samples up to a distance of 2 mm (Fig. 7). This effect was observed beyond the ‘surface/wall effect’ described above. These samples only differ by the ratio of root volume to visible porosity (Fig. 8), i.e. if there are enough macropores of the appropriate size available in relation to root length density and their diameter class distribution, roots do not necessarily need to create new pores. These findings are in line with research on root soil contact, i.e. root-soil contact decreases if a connected macropore system exists^5,37,38^.

Only for intermediate bulk density along with relative high root length densities compaction of the rhizosphere as described by Dexter^12^ was observed. The decreases of visible porosity (Fig. S2) by more than 25% is in line with findings of Bruand *et al*.^11^, who observe a reduced porosity of 22–24% around maize roots.

If we transfer all these findings to the field, we can suggest following processes, which led to the differences in compaction around biopores in the field:

1. Directly after reclamation the roots of Lucerne found sufficient macropores and therefore the rhizosphere of the created biopores remained highly porous and no changes in pore size distribution in 40–60 cm were observed. At later time points roots could not find sufficient empty connected macropores which led to an increase in root-induced compaction over time (Fig. S4).
2. Due to tillage in the topsoil on the 6, 12 and 24 year old fields some biopores were destroyed, but visible porosity increased. Roots of the current crop therefore could use these new available macropores created by tillage, which facilitated a more porous rhizosphere compared to the bulk soil underneath the plough layer. However, Lucas *et al*.^7^ also found that on tilled fields a lower amount of connections occurred. If roots therefore grow into existing dense clods, they needed to create new channels while compacting the surrounding soil. These two processes may have led to the high standard deviation in this depth (Fig. S5).

The compaction was mainly visible around roots >250 µm in the lap experiment (Fig. 7), which is in line with the model of Dexter^12^. In addition, small roots (<250 µm) were more likely to find existing pores of the appropriate size, since sufficient pores larger than the root diameter were available (Fig. 8). This is in line with Bodner *et al*.^39^ who showed that especially plants with coarse root types have increase macropore volume while roots of fine rooting types only have an impact on micropores, since they can use existing macropores^39^.

However, the resulting densities around coarse and fine roots in the samples of the lowest bulk density showed a contradictory result. The only explanation for this is that fine roots in these samples predominantly grew into more dense areas. Potentially such a behavior could have been triggered by secondary factors such as water or nutrient availability. Large pores are drained first during drying, this can induce laterals to form resulting in so called ‘hydropatterning’^40^.

These differences in rhizosphere physics cause dramatic differences in hydraulic properties of the biopore walls^9,10^. A high density, such as in the rhizosphere of roots in 1.45 g cm^−3^ samples, leads to high mechanical resistance. The maximum density (gray values) in the rhizosphere of these samples is similar to that of the bulk soil in the 1.60 g cm^−3^ samples (Fig. 5). Since the density of 1.60 g cm^−3^ at the given water level already strongly limited root elongation it is likely that roots can also not elongate into the compacted rhizosphere of roots in 1.45 g cm^−3^ treatment. If we transfer this to the field, roots growing into biopores on the 12-year old soil, with similarly compacted walls, may be trapped. This would be in line with findings that growing roots can be trapped within biopores^3^, especially in more compacted soil layers^41^. Lucas *et al*.^7^ calculated the Euclidean distances between the biopores and showed that with the high biopore length densities on the 12-year old field, the distance between biopores is typically around 2–3 mm. If we consider a compacted area which reaches up to 1 mm into soil, this means one dense zone borders on the next. A new root can therefore only grow within the biopore or right in the middle of two biopores. The later would probably lead to destruction of the old biopores since the new root also align the particles radial up to 1 mm distance. This all could be the possible explanation of the maximum of biopore densities found by Lucas *et al*.^7^ that reached a plateau already half-way through the chronosequence.

## Supplementary information

Supplementary Information
