# Supplementary material for: Roots compact the surrounding soil depending on the structures they encounter
Source: Sci Rep. 2019 Nov 7;9:16236. doi: 10.1038/s41598-019-52665-w (PMC6838105; doi:10.1038/s41598-019-52665-w)
Supplement: Supplementary file 1 — Supplementary Information [file 41598_2019_52665_MOESM1_ESM.pdf]

Supporting Information

Article title: Roots compact the surrounding soil depending on the structures they encounter
Authors: Maik Lucas, Steffen Schlüter, Hans-Jörg Vogel, Doris Vetterlein

The following Supporting Information is available for this article:

Table S1: Mean values and standard error of plant dry weight and measured nutrients. Different letters indicate significant differences between treatments.

Fig. S1: Frequency histograms of the euclidean distance from soil voxels to the next root for each of the three different treatments (bulk density of 1.30, 1.45 and 1.60 g cm^{-3}) and depth within column (7 – 10 cm, 12-15 cm, 17-20 cm).

Fig. S2: Mean change in visible porosity with the distance to the root surface relative to the mean visible porosity of a sample.

Fig. S3: Mean change in macroporosity with the distance to the root surface of different root diameters relative to the mean macroporosity of a sample.

Fig. S4: Mean volume of biopore and pore diameter classes of all field samples.

Fig. S5: Mean change in gray value with the distance to the biopore surface relative to the mean gray value of a sample for all field samples

Table S1: Mean values and standard error of plant dry weight and measured nutrients. Different letters indicate significant differences between treatments.

	**Treatment**					
	1.30 [g cm^{-3}]		1.45 [g cm^{-3}]		1.60 [g cm^{-3}]	
**Plant dry weight [g]**	0.69^{a}	±0.06	0.55^{a}	±0.06	0.24^{b}	±0.04
**C [mg g^{-1}]**	418.41^{a}	±1.02	418.45^{a}	±1.27	416.90^{a}	±2.39
**N [mg g^{-1}]**	28.60^{a}	±0.61	30.25^{a}	±0.59	30.15^{a}	±0.46
**C/N-ratio [-]**	14.66^{a}	±0.34	13.89^{a}	±0.34	13.83^{a}	±0.30
**P [mg g^{-1}]**	1.83^{a}	±0.04	1.74^{ab}	±0.05	1.59^{b}	±0.05
**K [mg g^{-1}]**	26.85^{a}	±0.61	28.46^{a}	±0.63	17.66^{b}	±0.55
**Ca [mg g^{-1}]**	15.51^{ab}	±0.77	14.45^{b}	±0.80	20.02^{a}	±0.63
**Mg [mg g^{-1}]**	5.13^{a}	±0.07	4.39^{a}	±0.23	4.90^{a}	±0.23
**Mn [µg g^{-1}]**	71.99^{a}	±2.68	68.05^{a}	±2.54	42.54^{a}	±2.10
**Fe [µg g^{-1}]**	93.38^{ab}	±3.94	105.29^{a}	±4.12	89.78^{b}	±5.05

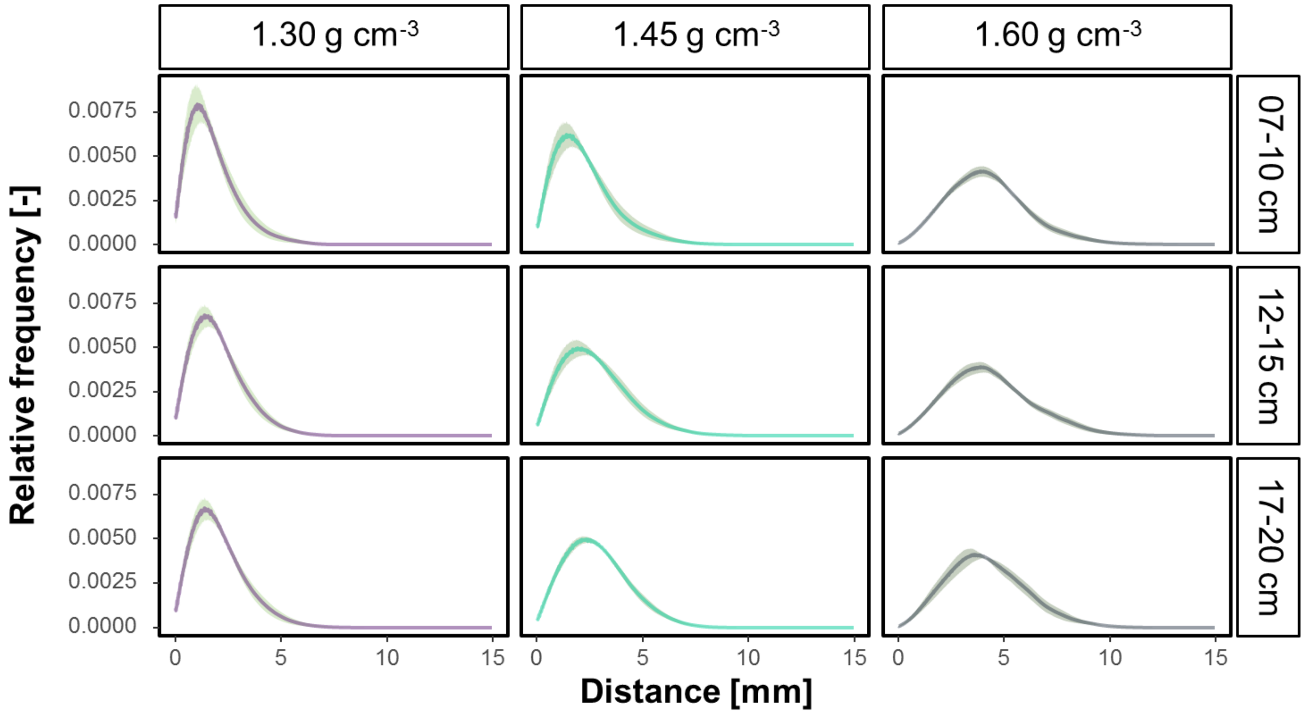

Fig. S1: Frequency histograms of the euclidean distance from soil voxels to the next root for each of the three different treatments (bulk density of 1.30, 1.45 and 1.60 g cm^{-3}) and depth within column (7 – 10 cm, 12-15 cm, 17-20 cm). Shadows indicate the standard error (n=5).

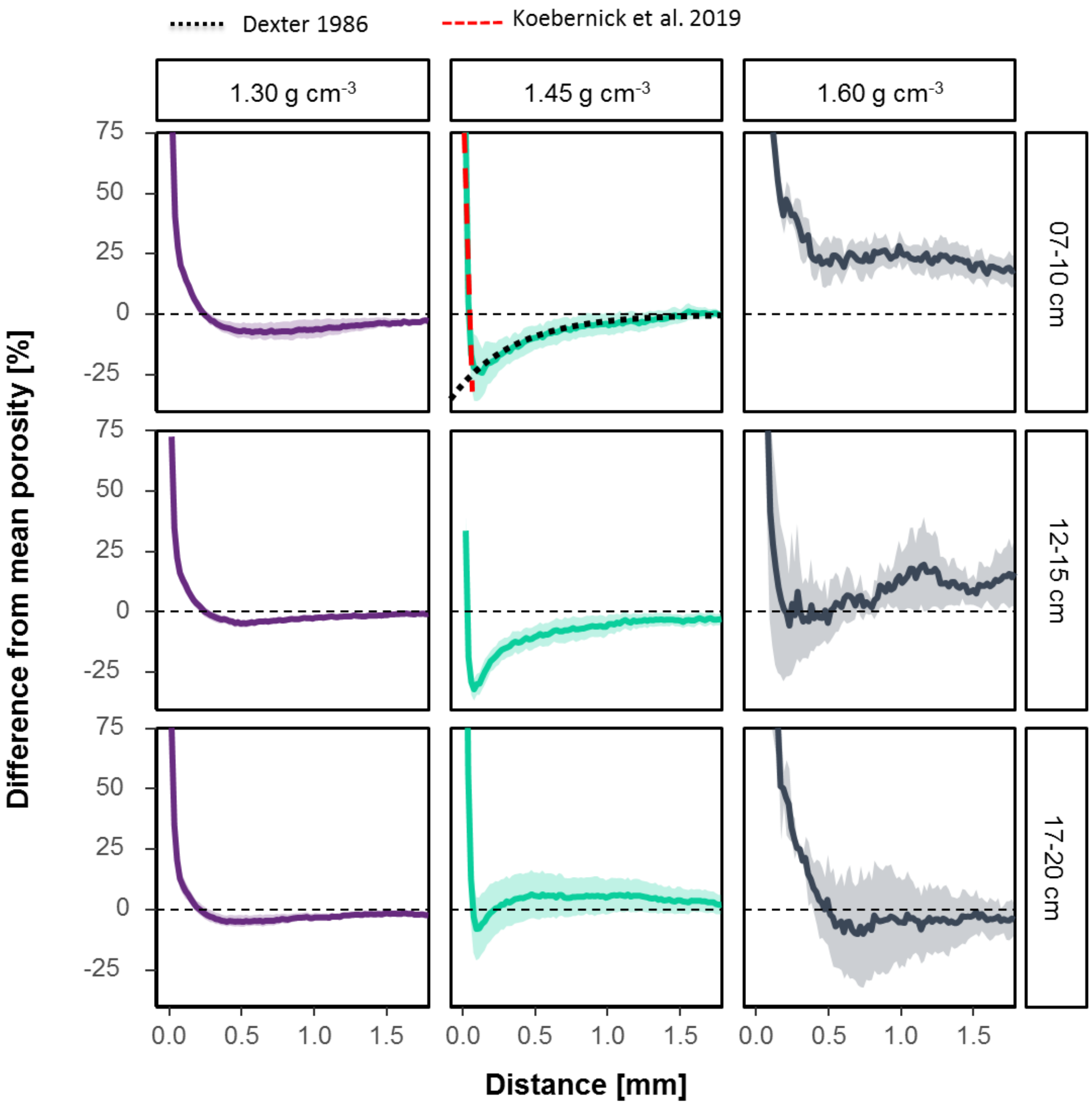

Fig. S2: Mean change in visible porosity with the distance to the root surface relative to the mean visible porosity of a sample. Shadows indicate the standard errors (n=5). For the first depth of the treatment with a bulk density of 1.45 g cm^{-3} the model of Dexter was used to calculate the exponential decrease of porosity at the root surface toward the bulk porosity. The same value for k (0.68) was used like in Dexter 1986, the mean root diameter and the porosity at the distance of the highest compaction and mean porosity in 07 – 10 cm of the treatment were used. The model of Koepernick et al. (2019) was fitted accordingly for only the points starting at the direct vicinity of the epidermis up to the point of highest compaction with a NLS in R. For this model only two parameters were unknown, the constant δ and the particle diameter. This resulted in a particle diameter of 1.974 mm, which corresponds to the 2 mm sieving.

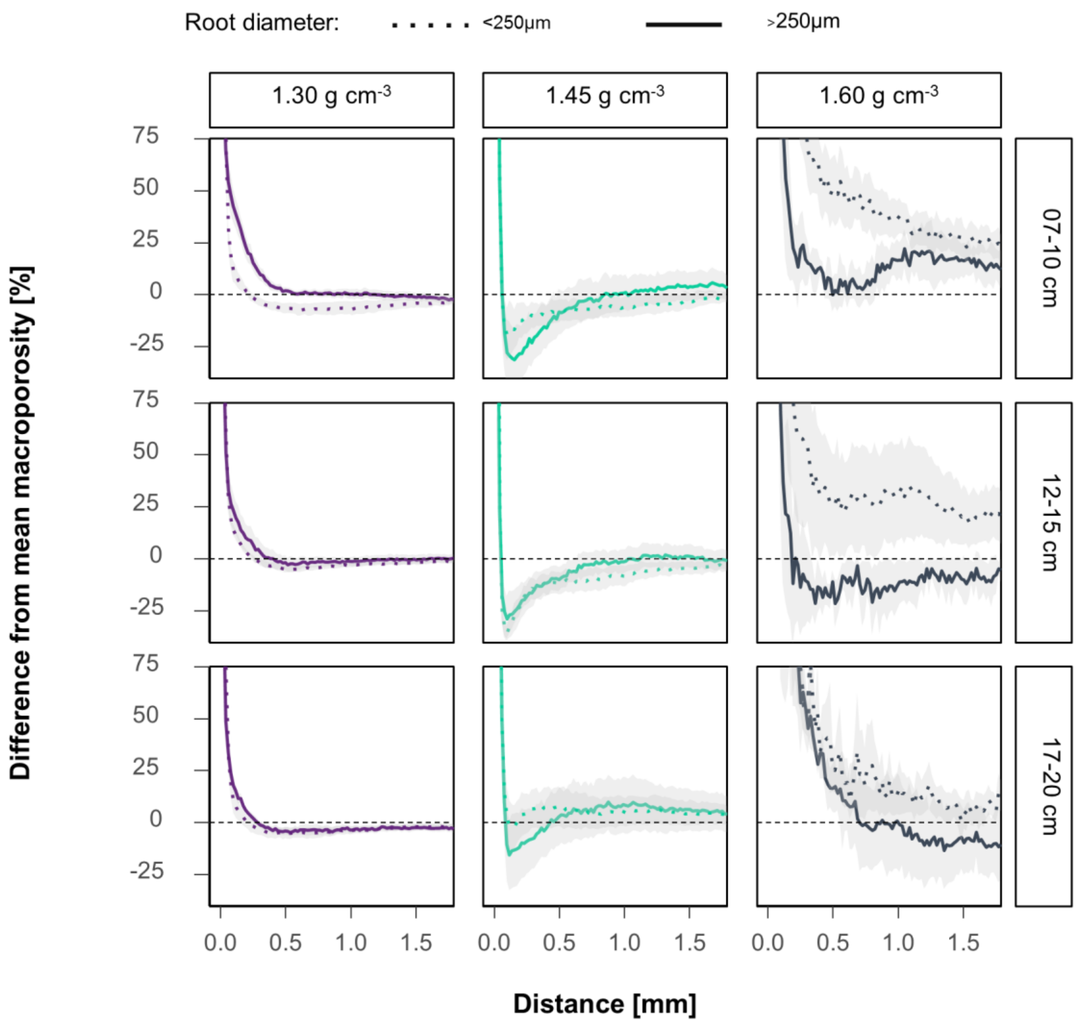

Fig. S3: Mean change in macroporosity with the distance to the root surface of different root diameters relative to the mean macroporosity of a sample. Dotted lines represent the mean changes around roots smaller 250 µm and solid lines these for roots with diameters greater than 250 µm. Shadows indicate the standard errors (n=5).

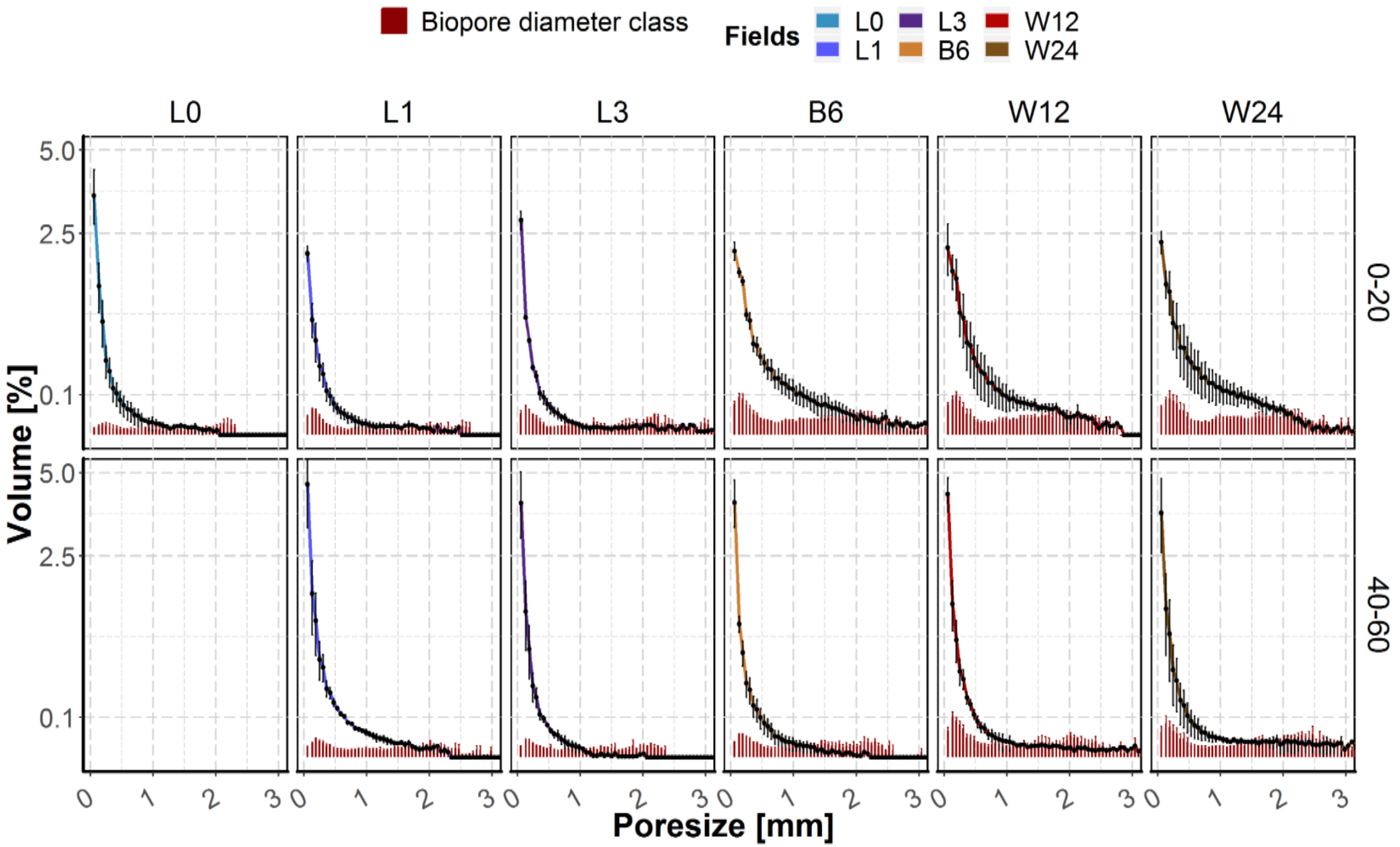

Fig.S4: Mean volume of biopore and pore diameter classes of all field samples. L0, L1, L3 were lucerne sites (0, 1, 3 years after reclamation). B6 was a barley field (6 years after reclamation)

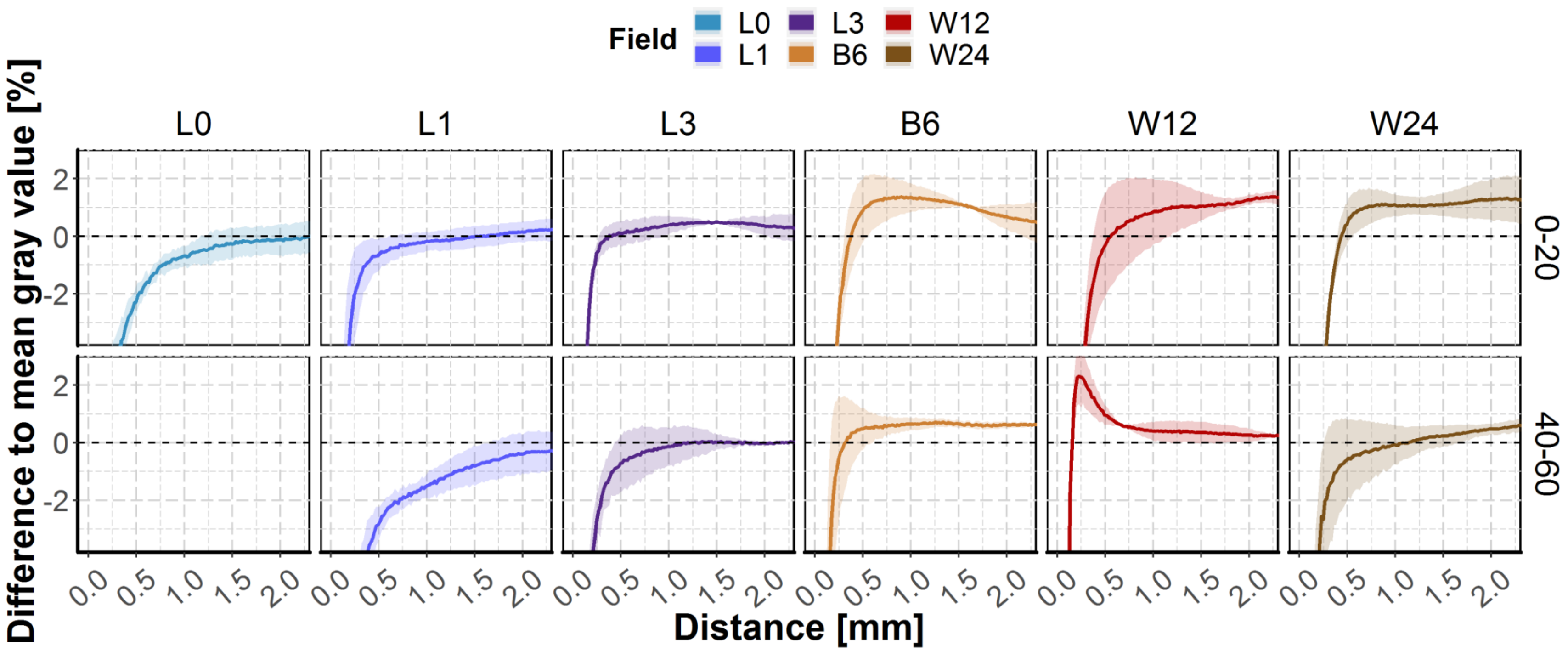

Fig. S5: Mean change in gray value with the distance to the biopore surface relative to the mean gray value of a sample for all field samples. L0, L1, L3 were Lucerne sites (0, 1, 3 years after reclamation). B6 was a barley field (6 years after reclamation) and W12 and W24 wheat fields (12 and 24 years after reclamation). Shadows indicate the standard deviation (n = 3 plots, which in turn each are determined by the result of 9 µCT scans (L3: n= 2).
